# Supplementary material for: Interpretable integration of unpaired multi-omics for Alzheimer’s diagnosis via cross-modal transformer reconstruction
Source: PLoS Comput Biol. 2026 Mar 12;22(3):e1014074. doi: 10.1371/journal.pcbi.1014074 (PMC12994821; doi:10.1371/journal.pcbi.1014074)
Supplement: S1 Table — (DOCX) [file pcbi.1014074.s001.docx]

Supplementary Table 1: Supplementary table for performance comparison of AE-Trans and other methods

| Methods |  | Accuracy | Precision | Recall | F1-measure | AUC |
| --- | --- | --- | --- | --- | --- | --- |
| RF | CV | 0.8743 | 0.9428 | 0.8592 | 0.8941 | 0.9424 |
|  | Test | 0.8418 | 0.9376 | 0.8083 | 0.8682 | 0.9352 |
|  | External | 0.6462 | 0.6336 | 0.7304 | 0.6786 | 0.6869 |
| NB | CV | 0.8486 | 0.8997 | 0.8611 | 0.8794 | 0.8499 |
|  | Test | 0.8755 | 0.9236 | 0.8795 | 0.9010 | 0.8739 |
|  | External | 0.6808 | 0.6461 | 0.8306 | 0.7269 | 0.6817 |
| LR | CV | 0.8166 | 0.9632 | 0.7439 | 0.8374 | 0.9533 |
|  | Test | 0.8080 | 0.8035 | 0.9455 | 0.8067 | 0.8463 |
|  | External | 0.7089 | 0.8622 | 0.2799 | 0.4226 | 0.7877 |
| AE-XGBoost | CV | 0.9178 | - | - | - | 0.9382 |
|  | Test | 0.8994 | 0.8934 | 0.9583 | 0.9247 | 0.9436 |
|  | External | 0.5113 | 0.5113 | 0.9032 | 0.6767 | 0.5660 |
| DEG-DMP-DNN | CV | 0.7924 | 0.9181 | 0.7400 | 0.8109 | 0.8139 |
|  | Test | 0.7896 | 0.9379 | 0.7212 | 0.8154 | 0.8173 |
|  | External | 0.6026 | 0.6204 | 0.5738 | 0.5962 | 0.6032 |
| Deep belief | CV | 0.9002 | 0.9081 | 0.9420 | 0.9245 | 0.9138 |
|  | Test | 0.8818 | 0.8806 | 0.9447 | 0.9115 | 0.9028 |
|  | External | 0.7161 | 0.7109 | 0.7497 | 0.7298 | 0.7652 |
| AE-Trans | CV | 0.9571 | 0.9749 | 0.9582 | 0.9664 | 0.9883 |
|  | Test | 0.9736 | 0.9788 | 0.9803 | 0.9796 | 0.9910 |
|  | External | 0.7389 | 0.6901 | 0.8884 | 0.7768 | 0.8432 |
