# Supplementary material for: Interpretable integration of unpaired multi-omics for Alzheimer’s diagnosis via cross-modal transformer reconstruction
Source: PLoS Comput Biol. 2026 Mar 12;22(3):e1014074. doi: 10.1371/journal.pcbi.1014074 (PMC12994821; doi:10.1371/journal.pcbi.1014074)
Supplement: S2 Table — (DOCX) [file pcbi.1014074.s002.docx]

Supplementary Table 2: Results obtained by different feature selection methods

| Methods | Feature Number | Accuracy | Precision | Recall | F1-measure | AUC |
| --- | --- | --- | --- | --- | --- | --- |
| Random | 200 | 0.4208 | 0.5536 | 0.5234 | 0.5381 | 0.3510 |
| F-score | 200 | 0.6528 | 0.7377 | 0.7159 | 0.7266 | 0.6663 |
| CV2 | 200 | 0.4072 | 0.5502 | 0.4393 | 0.4886 | 0.3375 |
| PCA | 200 | 0.5395 | 0.6790 | 0.5416 | 0.6026 | 0.5356 |
| AE-Trans | 200 | 0.8999 | 0.9704 | 0.8712 | 0.9181 | 0.9749 |
| DEG-DMP | 200 | 0.8579 | 0.9349 | 0.8378 | 0.8837 | 0.9359 |
