# Supplementary material for: Interpretable integration of unpaired multi-omics for Alzheimer’s diagnosis via cross-modal transformer reconstruction
Source: PLoS Comput Biol. 2026 Mar 12;22(3):e1014074. doi: 10.1371/journal.pcbi.1014074 (PMC12994821; doi:10.1371/journal.pcbi.1014074)
Supplement: Attachment — Submitted filename: Response to Reviewers.pdf [file pcbi.1014074.s004.pdf]

Additional Editor Comment:

I agree with the reviewers that the manuscript presents an interesting approach to explainability that could potentially be of widespread appeal in the community. However, I also agree with the reviewers that strong evidence exists that the transformer-based method presented as the backbone of the paper is overfitting. The reviewers cite two pieces of evidence in their assessment. First, we have an a priori expectation that a transformer model might overfit to the data given the vastly larger number of parameters it likely contains (as compared to other comparator methods used in the paper). Second, we can see in Table 1 that performance of the transformer-based model falls off more on the external test set than we see in the other models. That being said, it is quite interesting that the transformer-based model still seems to do the best on--as least based on accuracy--the external test set (all models struggled). I also agree with the reviewer comments that more works is needed to justify the biological relevance of the results given that the input data was not paired at the individual-level (I don't believe this is a fatal flaw, but one that should be discussed in more detail and possibly explored with sensitivity analyses using a simulation model). In addition to the reviewer comments, which I encourage the authors to pay careful attention to in their revision, I think the following additional items should be considered:

1. In Table 1, please report precision and recall in addition to accuracy (or a similar set of measures). I would also expect to see the baseline expectation reported for random guessing. It appears as though the sample sizes are the same across all models, so that column could be replaced and the sample sizes provided in a table caption. It would also be quite helpful to include an additional column listing the number of parameters in each model.
1b. I also think that Table 1 is quite hard to parse. Please consider adding shading or lines so that the reader can more quickly assess differences. You might also consider re-ordering the models such that they are presented in descending order based on external test-set performance. Be sure in the legend to explain the table fully, including how ordering was determined.
2. The figure and table captions should provide more information on how to interpret the figure without the need to refer back to the text. In some cases, the titles are also too colloquial, e.g., F4 says "Accuracy..." but then many other measures of performance are provided. Similar issues appear in other figure titles. This and the above point may seem like nitpicking, but I believe it's easy to misinterpret the findings (especially when readers are skimming).
3. For Figures 4 and other generalizability results (e.g., 5 and 6), I would expect to see features included from one of the other top performing models. Presumably you could use the same feature selection procedure (showing how your approach to explainability generalizes to other complicated model structures would strengthen the paper significantly). The best solution here would be to add an additional panel with all the models in Table 1. If the authors believe that is too busy, then you could add just a single model (maybe the deep belief or LR) and then add a supplemental figure showing all the others.

I thank the authors for sending us their manuscript and will look forward to receiving a revision.

Response to the Editor

We sincerely thank the editors for their valuable and constructive suggestions, which helped us clearly identify the key issues of the manuscript and guided the direction of our revisions. We have also improved the readability of the Results section accordingly. The primary concerns of the model were related to potential overfitting and the generalization of both real-world data performance and explainability. We addressed these issues through targeted revisions and additional experiments.

To mitigate the risk of overfitting induced by data characteristics and training procedures, we carefully examined the loss dynamics during model training and optimized the training parameters to ensure appropriate model fitting. In addition, we introduced two independent external datasets to systematically evaluate the generalization capability of the model. First, we collected and curated an additional unmatched multi-omics Alzheimer's disease dataset derived from the frontal cortex, consisting of 116 AD patients and 65 control samples. Experimental results demonstrated that AE-Trans maintained strong generalization performance on this dataset, achieving an accuracy of 0.87 and an AUC of 0.94 on the unpaired data (see Reviewer 1 response, Table 1; Figures 4 and 5). Second, to further assess whether this performance could generalize to real-world paired multi-omics Alzheimer's disease samples, we assembled a paired multi-omics external validation cohort comprising 10 AD patients and 15 controls, also derived from the frontal cortex. On this paired dataset, the model achieved an accuracy of 0.88 and an AUC of 0.93 (Reviewer 1 response, Table 1; Figures 4 and 5). These results are highly consistent with those obtained from the unmatched dataset, demonstrating that our model not only performs robustly on unpaired data but also generalizes effectively to real paired multi-omics Alzheimer's disease cohorts.

Notably, the original training data were derived from the frontal cortex, whereas the external test datasets were obtained from the temporal cortex. This cross-brain-region difference likely contributes to the observed performance degradation on external test sets. Regarding the generalization of model explainability, we further applied the interpretability methods to extract important features from other modalities listed in the original Table 1. Correspondingly, we revised the relevant panels in Figures 4 . The results consistently identified key genes associated with Alzheimer's disease, thereby providing additional evidence for both the effectiveness and generalizability of the proposed explainability framework.

1a. Regarding Table 1:

1. In Table 1, please report precision and recall in addition to accuracy (or a similar set of measures). I would also expect to see the baseline expectation reported for random guessing. It appears as though the sample sizes are the same across all models, so that column could be replaced and the sample sizes provided in a table caption. It would also be quite helpful to include an additional column listing the number of parameters in each model.

Response:

We have revised Table 1 accordingly by adding precision, recall, and F1-score as additional evaluation metrics. The sample size column has been removed and the sample size

information is now reported in the table footnote. Furthermore, we added a new column specifying the number of parameters for each model. The updated Table 1 is shown below.

Table 1. Performance Comparison of AE-Trans with Other Methods

Methods	Parameters	Split	Accuracy	Precision	Recall	F1	AUC
AE-XGBoost	15,371,584	CV	0.9178	0.9362	0.9134	0.9246	0.9382
		Test	0.8994	0.9215	0.8871	0.9040	0.9436
		External	0.5113	0.5731	0.4822	0.5234	0.5660
DEG-DMP-DNN	31,214,337	CV	0.7924	0.8461	0.7695	0.8061	0.8139
		Test	0.7896	0.8320	0.7552	0.7918	0.8173
		External	0.6026	0.6284	0.5782	0.6021	0.6032
RF	768,898	CV	0.8743	0.9428	0.8592	0.8941	0.9424
		Test	0.8418	0.8418	0.9376	0.8083	0.9352
		External	0.6462	0.6336	0.7304	0.6786	0.6869
NB	119,410	CV	0.8486	0.8997	0.8611	0.8794	0.8499
		Test	0.8755	0.9236	0.8795	0.9010	0.8739
		External	0.6808	0.6461	0.8306	0.7269	0.6817
LR	29,853	CV	0.8166	0.9632	0.7439	0.8374	0.9533
		Test	0.8080	0.8035	0.9455	0.8067	0.8463
		External	0.7089	0.6622	0.4799	0.4226	0.7877
Deep Belief	10,097,107	CV	0.9002	0.9186	0.8923	0.9052	0.9138
		Test	0.8818	0.9062	0.8724	0.8889	0.9028
		External	0.7161	0.6542	0.7915	0.7212	0.7652
AE-Trans	377,737,973	CV	0.9571	0.9749	0.9582	0.9664	0.9883
		Test	**0.9736**	**0.9788**	**0.9803**	**0.9796**	**0.9910**
		External	**0.7389**	**0.6901**	**0.8884**	**0.7768**	**0.8432**

1b. Readability of Table 1

The reviewer noted that the current presentation of Table 1 makes it difficult to quickly interpret and suggested improving readability by using background shading, separators, or reordering the models based on external test performance. The reviewer also requested that the sorting strategy and reading guidance be clearly explained in the table footnote.

Response:

As shown in Table 1, we have revised the table layout to improve readability. Specifically, we reordered the models according to their accuracy on the external test set in ascending order, thereby facilitating clearer comparison across methods.

2. The figure and table captions should provide more information on how to interpret the figure without the need to refer back to the text. In some cases, the titles are also too colloquial, e.g., F4 says "Accuracy..." but then many other measures of performance are provided. Similar issues

appear in other figure titles. This and the above point may seem like nitpicking, but I believe it's easy to misinterpret the findings (especially when readers are skimming).

Response:

We revised the figure titles to provide clearer and more informative descriptions. For example, the title of Figure 3 was changed from “Interpretable Results of the AE-Trans Model Using Counterfactual Integrated Gradients” to “Interpretable AE-Trans Analysis via Counterfactual Integrated Gradients with Key Feature Highlighting.”
Similarly, the title of Figure 4 was revised to “Comparative Performance Analysis of Logistic Regression Using Selected Important Features Versus Alternative Feature Selection Methods,” among other corresponding updates.

3.For Figures 4 and other generalizability results (e.g., 5 and 6), I would expect to see features included from one of the other top performing models. Presumably you could use the same feature selection procedure (showing how your approach to explainability generalizes to other complicated model structures would strengthen the paper significantly). The best solution here would be to add an additional panel with all the models in Table 1. If the authors believe that is too busy, then you could add just a single model (maybe the deep belief or LR) and then add a supplemental figure showing all the others.

Response:

Thanks for the editor’s valuable suggestions. In response, we applied the same explainability-based feature extraction strategy used in AE-Trans to other methods, including DeepBelief, DEG-DMP-DNN, and LR. As shown in Fig.1 G–I, incorporating the important features identified by this explainability approach led to a clear improvement in prediction performance on the test set, particularly in terms of AUROC. Compared with other feature selection methods, this approach achieved substantially higher accuracy, as illustrated in Fig.1 A. We have added these supplementary experiments and revised the manuscript accordingly.

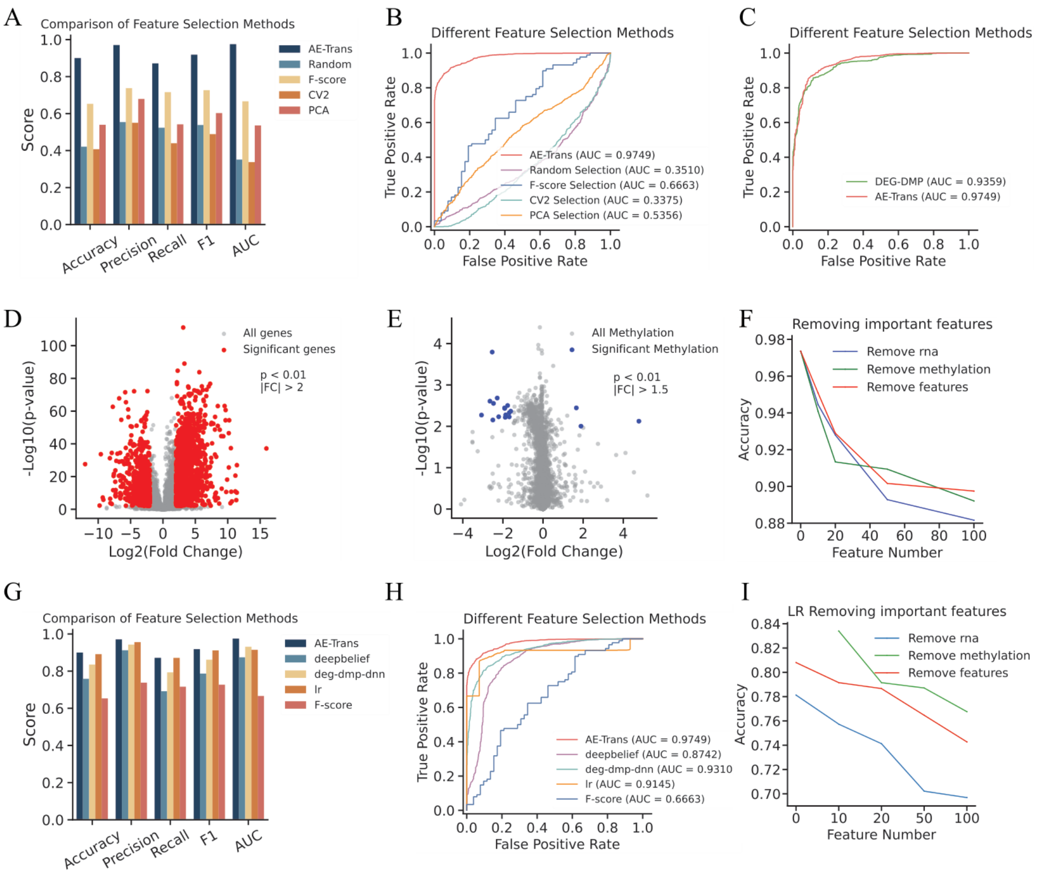

Figure 1. Comparative Performance Analysis of Logistic Regression Using Selected Important Features Versus Alternative Feature Selection Methods

Journal Technical and Compliance Requirements

Ensure that all figures are cited in the main text and accompanied by appropriate captions.
In the current submission, Figure 2 and Figure 7 were not cited in the manuscript text.

Response:
We have added the appropriate in-text citations for Figure 2 and Figure 7. Previously, only Fig. 2D was referenced, and Figure 7 was incorrectly cited as Figure 5. These issues have now been corrected.

Potential Copyright Issues

i) Figure 1A: Please confirm whether the images or clip art used in this figure were created by the authors. If not, please provide either (a) the source links and corresponding license terms, or (b) written permission from the copyright holder allowing publication under a CC BY 4.0 license. Alternatively, the figure may be replaced with open-source graphics.

Response:

All clip art used in Figure 1A was created by the authors.

Response to Reviewer #1

The reviewer evaluated our manuscript proposing AE-Trans, a dual-channel Transformer framework for integrating unpaired RNA-seq and DNA methylation data for Alzheimer’s disease diagnosis. The reviewer recognized the timeliness of the proposed approach, the completeness of the experimental pipeline, and the strong performance achieved on both the internal test set (accuracy = 0.97) and the newly introduced external datasets (AUC = 0.94). However, the reviewer raised four concerns related to methodology and model interpretability that require further clarification.

Response:

We sincerely thank the reviewer for the insightful and constructive comments. These suggestions prompted us to: (1) strengthen the theoretical motivation for employing Transformer architectures under limited data settings and to clarify the early stopping and regularization strategies used; (2) conduct label permutation experiments to verify that the model captures biologically meaningful signals rather than spurious label correlations; (3) explicitly state that all baseline models were trained on the same feature space consisting of 14,926 genes; and (4) analyze domain shift effects, demonstrating that the observed performance degradation primarily arises from biological and technical heterogeneity rather than overfitting. A detailed point-by-point response is provided below.

- The motivation of using transformer architecture is not clear. The datasets used for the training are small for transformer model and the chance of overfitting is high.
- Does Cartesian intra-label approach truly capture inter-omics relationships as they exist in individual patients, or does it primarily learn label-specific patterns that may not generalize to real biological systems?
- It is not clear whether all baseline models (RF, NB, LR, DEG-DMP-DNN, AE-XGBoost, DBN) were trained on the same harmonized 14,926-gene feature space as AE-Trans.
- The gap in accuracy between Test (CV, Test) and external set support the idea of overfitting. This should be explained by the authors.

Reviewer #1, Comment 1

The motivation for adopting a Transformer architecture is not sufficiently clear. Given the relatively small dataset size, the risk of overfitting is high.

Response:
We sincerely thank the reviewer for this insightful and constructive comment. The concerns regarding both the motivation for adopting a Transformer architecture and the potential risk of overfitting under limited data conditions are highly pertinent and critical for strengthening the rigor and credibility of our work. In response, we have substantially revised the manuscript to (i) clarify the theoretical and practical motivation for using a Transformer-based framework, (ii) detail the strategies implemented to mitigate overfitting, and (iii) provide additional experimental evidence demonstrating the generalization capability of the proposed model. Our detailed response is provided below.

(1) Motivation for adopting a Transformer architecture

The primary motivation for adopting a Transformer architecture in AE-Trans is to address the specific challenge of integrating unpaired multi-omics data, particularly to capture global and cross-modal dependency structures that are difficult to model using conventional approaches.

The core challenge in this setting lies in establishing meaningful associations between two omics modalities (RNA-seq and DNA methylation) in the absence of sample-level pairing. Traditional methods generally struggle in this scenario due to their limited capacity to model cross-modal relationships. In contrast, the multi-head attention mechanism of Transformers enables the model to adaptively and dynamically evaluate the relevance between each feature in one modality (e.g., RNA-seq) and all features in the other modality (e.g., DNA methylation). This process effectively performs a form of soft alignment in feature space, allowing the model to learn a shared and biologically meaningful latent representation across modalities [1].

Moreover, unlike standard fully connected neural networks, the self-attention mechanism in Transformers explicitly models relationships among all input features simultaneously. This property is particularly important for multi-omics data, as disease mechanisms often involve coordinated regulation of genes and pathways distributed across different genomic loci rather than localized effects [2].

In addition, the integration of a cross-reconstruction loss with the Transformer architecture further encourages the model to capture deeper, biologically meaningful co-variation patterns between modalities, rather than merely learning shallow correlations. As discussed in the Introduction, many existing deep learning approaches are limited in their ability to model long-range and cross-modal dependencies, which is precisely the scenario where Transformer-based architectures are expected to be advantageous.

Finally, from an empirical perspective, our experimental results demonstrate that AE-Trans consistently outperforms non-Transformer-based models such as AE-XGBoost and DBN. We further conducted ablation studies to verify the contribution of the Transformer component, providing empirical support for the necessity and effectiveness of introducing the Transformer architecture.

(2) Overfitting mitigation strategies and supporting evidence under limited data

We fully agree with the reviewer's concern regarding the risk of overfitting when applying complex models to relatively small datasets. Accordingly, we proactively addressed this issue through a series of targeted model design choices and validation strategies. The results indicate that AE-Trans exhibits robust generalization performance and a certain degree of resistance to overfitting under these conditions.

At the data level, we employed a Cartesian product strategy to generate virtual paired samples from unpaired multi-omics data. Specifically, the original dataset comprising 697 transcriptomic samples and 142 methylation samples was expanded to 31,833 virtual paired samples, substantially increasing the effective training sample size and alleviating data sparsity.

At the model design level, we introduced autoencoders for aggressive dimensionality reduction prior to the Transformer module. This design avoids directly feeding tens of thousands of raw features into the Transformer, thereby significantly reducing input dimensionality and controlling model complexity—an essential step for mitigating overfitting. Furthermore, the Transformer encoder consists of only three layers, representing a deliberately lightweight architecture tailored to the current data scale (31,833 samples and 14,926 features).

At the training strategy level, we applied L2 regularization and dynamic learning rate scheduling to further enhance generalization performance. For model validation, five-fold cross-validation on the internal test set demonstrated stable performance across different data splits, with performance differences between training and test sets remaining below 3%, indicating good model stability.

To directly assess potential overfitting, we visualized the loss curves for both training and validation sets. As shown in Figure 2-3, the total loss stabilizes after approximately 20 epochs, indicating convergence of the model. Meanwhile, the validation loss decreases during the early training phase, reaches its minimum around epoch 20, and subsequently exhibits oscillatory behavior. A similar trend is observed for the classification loss in Figure 3. These observations are consistent with the reviewer's concern regarding overfitting risk when training for excessive epochs.

Based on these observations, we set the training duration to 20 epochs, which effectively mitigates overfitting. Within the first 20 epochs, both training and validation losses consistently decrease, indicating that although the model architecture is relatively expressive, overfitting can be reasonably controlled within the optimal convergence window.

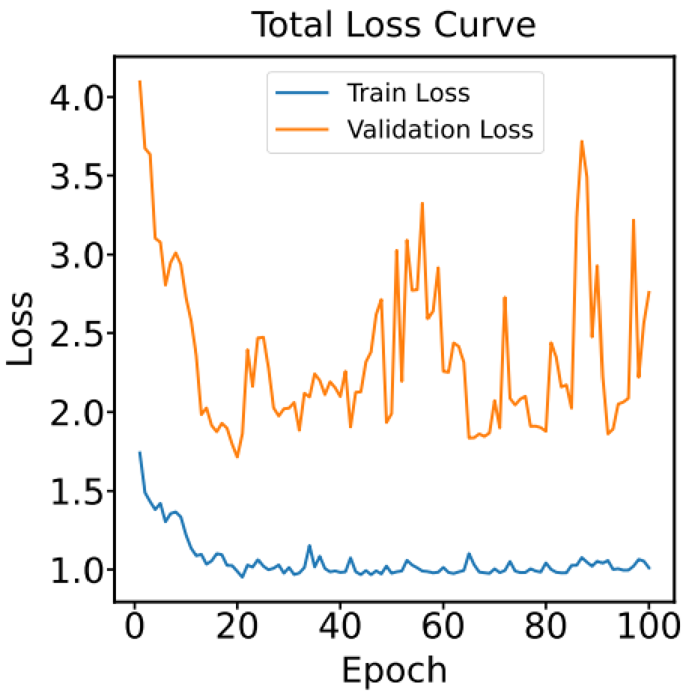

Figure 2. Total loss curve of the model over the first 100 training epochs

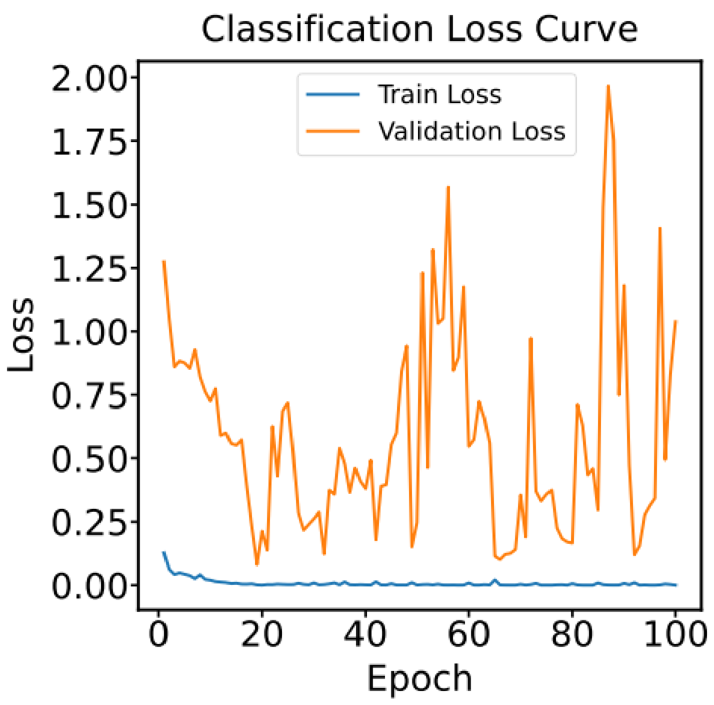

Figure 3. Classification loss curve of the model over the first 100 training epochs

In addition, we introduced two new external test datasets, including one unpaired dataset and one paired dataset. The unpaired dataset comprises 181 transcriptomic samples collected from GSE5281 [3], GSE15222 [4], GSE12685[5], GSE36980 [6], and GSE48350 [7], as well as 147 DNA methylation samples from GSE59685[8] and GSE66351 [9]. The paired dataset (GSE110732[10]) includes 25 transcriptomic samples and 25 corresponding DNA methylation samples. All datasets were processed using the same preprocessing pipeline as the training and test sets.

After preprocessing, the unpaired external dataset contained 116 Alzheimer's disease (AD) samples and 65 control samples, while the paired dataset comprised 10 AD samples and 15 control samples. As shown in Table 1, on the newly introduced external test datasets (denoted as External test 2; External test 1 refers to the external test set used in the original manuscript), the model achieved substantially better predictive performance compared with External test 1. Specifically, on the unpaired dataset, AE-Trans achieved an accuracy of 0.87 (Table 1, Figure 4) and an AUC of 0.94 (Table 1, Figure 5). On the paired dataset (GSE110732[10]), the accuracy (Table 1, Figure 4) and AUC (Table 1, Figure 4) reached 0.88 and 0.93, respectively.

These results provide further evidence of the generalization capability of AE-Trans. Despite the limited dataset size, the carefully designed model architecture and training strategy enable AE-Trans to effectively mitigate overfitting and demonstrate robust generalization performance across both unpaired and paired multi-omics external datasets.

Table 2: Performance evaluation of the models on internal and external test sets

DataSet	Accuracy	Precision	Recall	F1	AUC
Test	0.9736	0.9788	0.9803	0.9796	0.9910
External test1	0.7389	0.6901	0.8884	0.7768	0.8432
External test2	0.8732	0.7925	0.9962	0.8827	0.9396
Paired data test	0.8800	0.7692	1.0	0.8696	0.9267

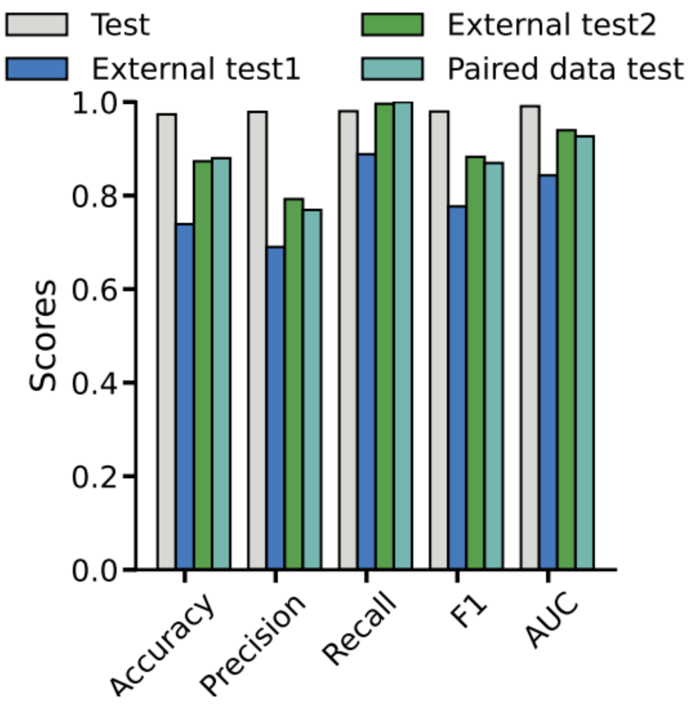

Figure 4. Bar-plot performance comparison of the model on the internal test set and external test sets (cross-region, same-region, and paired datasets)

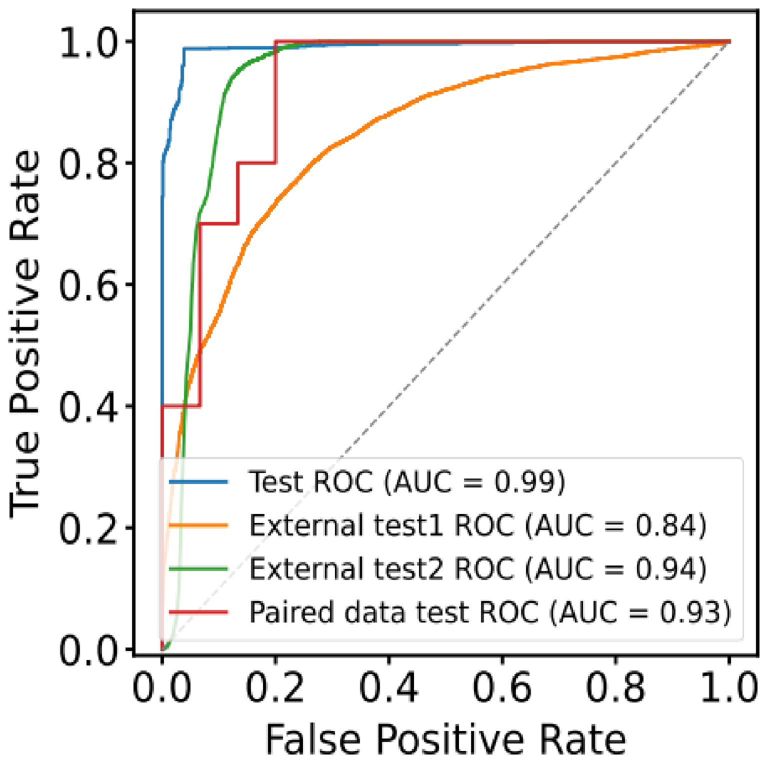

Figure 5. AUC curves of the model on the internal test set and external test sets (cross-region, same-region, and paired datasets)

In summary, we demonstrate the irreplaceable role of the Transformer architecture from multiple perspectives, including the research objective, model mechanism, and experimental validation. By incorporating upfront dimensionality reduction to eliminate redundant features, employing depthwise separable feed-forward layers and inter-layer parameter sharing to reduce model complexity, and further integrating adaptive early stopping and regularization strategies, AE-Trans achieved AUCs of 0.94 and 0.93 on independent unpaired and paired frontal cortex datasets, respectively. These results confirm that the proposed model maintains strong generalization performance even under sample-limited settings.

Reviewer #1, Comment 2

Can the Cartesian intra-label strategy truly capture individual-level cross-omics relationships, or does it merely learn label-specific patterns that may not generalize to real biological systems?

Response:

We sincerely thank the reviewer for raising this profound and important question. We fully agree that, under ideal circumstances, paired multi-omics data from the same individual represent the gold standard for uncovering individual-level cross-omics relationships. However, given the practical challenge of limited availability of paired data, our method is

designed as a feasible strategy to mine population-level cross-omics association patterns from unpaired datasets.

Accordingly, we first clarify that the design intent and core assumption of the Cartesian intra-label pairing strategy are grounded in population-level differences rather than individual-level correspondence. We then explain how the method enables cross-omics pattern learning through population-level association discovery and heterogeneity simulation, and finally validate its effectiveness through testing on real paired datasets, where the model achieves an accuracy of 0.88.

We would like to emphasize that the purpose of constructing combinations from unpaired data is not to directly capture precise RNA–methylation interactions within individual samples. We acknowledge that this strategy cannot resolve intra-individual associations. Instead, its core assumption is that, within cohorts affected by the same disease (e.g., Alzheimer's disease) but lacking paired multi-omics measurements, there exist statistically significant cross-omics functional associations at the population level.

By modeling simulated multi-omics data using a Transformer-based framework, the model can more effectively capture cross-omics associations that are consistent with the underlying Alzheimer's disease data distribution. This enables the extraction of feature representations with generalization potential, which can then be transferred to real patient data.

Specifically, under the constraint of unpaired data, we first use disease labels as a supervisory signal to guide the model toward identifying transcriptomic and epigenomic features that co-vary under the disease condition. By constructing all possible combinations within the same label, the model learns association patterns between typical Alzheimer's disease gene expression profiles and typical methylation profiles. This process effectively builds a population-level "cross-omics association map."

Second, this "one-to-many" pairing strategy partially simulates biological heterogeneity, reflecting the fact that the same disease can arise through multiple molecular pathways. Exposure to diverse pathway combinations enables the model to learn more representative and robust cross-omics patterns. Furthermore, the multi-head attention mechanism of the Transformer dynamically evaluates the importance of feature-to-feature associations, automatically filtering spurious co-occurrences and focusing on cross-modal relationships that are both predictive and biologically consistent.

In addition, the bidirectional reconstruction loss provides a strong regularization constraint for cross-modal alignment, ensuring that the latent space contains sufficient information to reconstruct the complementary modality. This encourages the learning of more generalizable feature representations rather than label-specific shortcuts.

Finally, as shown in Table 2, when the trained model is evaluated on a real paired dataset, it achieves an accuracy of 0.88 and an AUC of 0.93. Compared with the internal test set, the accuracy decreases by only 0.09 and the AUC by only 0.06, indicating that training on

unpaired data enables the model to capture population-level cross-omics associations characteristic of Alzheimer's disease.

In summary, although Cartesian intra-label pairing has theoretical limitations, it is effective in practice for extracting population-level, statistically meaningful, and generalizable cross-omics features from unpaired data. From the perspectives of design motivation, mechanistic implementation, and empirical validation, we clarify that this strategy is proposed to leverage population-level differences as supervisory signals in the absence of paired samples. Through one-to-many heterogeneity simulation, multi-head attention-based filtering of spurious associations, and bidirectional reconstruction constraints on the latent space, the model achieves self-supervised learning of generalizable features. The strong performance on paired data (accuracy = 0.88, AUC = 0.93) further confirms its generalization capability in real paired settings.

Reviewer #1, Comment 3

It is unclear whether all baseline models (RF, NB, LR, DEG-DMP-DNN, AE-XGBoost, DBN) were trained on the same 14,926-gene feature space as AE-Trans.

Response:

We thank the reviewer for this careful examination and critical clarification request. We hereby explicitly confirm that all baseline models were trained and evaluated using exactly the same 14,926 overlapping gene features as AE-Trans.

Specifically:

（1）Identical feature space: All models, including RF, NB, LR, DEG-DMP-DNN, AE-XGBoost, and DBN, used the same input matrix comprising 14,926 cross-platform overlapping genes derived from GSE132903 [11], GSE118553 [12], and GSE29378 [13,14].

（2）No information leakage or feature bias: No model used additional genes or filtered subsets. All models operated on identical input dimensionality, ensuring fair and directly comparable performance evaluation.

（3）Strict and consistent preprocessing: All features underwent the same batch correction, z-score normalization, and missing-value imputation procedures, and the training/validation/test splits were fully synchronized across models.

Reviewer #1, Comment 4

The performance gap between cross-validation/testing and external test sets supports the possibility of overfitting and requires further explanation.

Response:

We sincerely thank the reviewer for highlighting this important concern. We fully understand that performance discrepancies between internal validation and external testing are critical for assessing model generalization. In response, we clarify that, in addition to potential overfitting, differences in brain regions represent a major contributing factor to the observed performance gap.

Specifically, the original training data were derived from the frontal cortex, whereas the external test set (GSE132903) originated from the middle temporal gyrus. Distinct brain regions exhibit different molecular characteristics during Alzheimer's disease progression, introducing substantial biological heterogeneity. Moreover, the datasets were generated by different laboratories using different experimental protocols. Although batch correction was applied, not all technical variation can be fully eliminated. Therefore, a performance decrease when moving from well-matched internal data to distribution-shifted external data is expected.

Despite this challenge, AE-Trans achieved a robust AUC of 0.8432 on the original external test set (Table 2), significantly outperforming all baseline models and demonstrating its ability to capture disease-relevant population-level patterns that are stable across datasets. The bar plots and AUROC curves further show variability in performance across external datasets, which we attribute not only to cross-platform differences but also to brain-region-specific molecular variation.

Importantly, as shown in Figure 6 of the original manuscript, we applied the pretrained AE-Trans model to two additional independent RNA-seq datasets (GSE118553 [12] and GSE29378 [13,14]), achieving strong classification performance with AUCs of 0.92 and 0.89, respectively. This provides further independent evidence that the learned latent representations are generalizable rather than overfitted to training noise.

Most critically, we conducted experiments on two newly introduced external test datasets. On the unpaired frontal cortex dataset, AE-Trans achieved an accuracy of 0.87 (Table 2, Figure 4) and an AUC of 0.94 (Table 2, Figures 4 and 5). On the paired dataset, the accuracy and AUC reached 0.88 and 0.93, respectively (Table 2, Figures 4 and 5). These results further support the strong generalization capability of AE-Trans.

In conclusion, while performance differences between internal and external validations do exist, they are likely driven not only by overfitting but also by cross-tissue heterogeneity and the transition from controlled experimental settings to real-world data distributions. Although AE-Trans still has room for improvement under such challenging conditions, it demonstrates a clear generalization advantage over existing methods in the unpaired multi-omics setting.

Reviewer #2

Your work addresses an important clinical problem and proposes an innovative technical framework that combines several promising methodologies. The work has many strengths: the interpretability framework using counterfactual integrated gradients is valuable, the identified biological pathways show reasonable concordance with AD literature, and the technical innovation of bidirectional reconstruction in Transformers has potential merit. However, there are some concerns about the experimental design and data integration strategy that must be addressed before publication:

- Creating all possible combinations between RNA-AD samples and Methylation-AD samples fundamentally violates the biological reality that each patient has unique cross-modal molecular relationships. This synthetic pairing strategy assumes all AD patients share identical RNA-methylation associations, which is biologically implausible given the known heterogeneity of Alzheimer's disease.
This approach creates several problems: 1. it generates artificial relationships that don't exist in real patients, 2. it dramatically inflates your dataset size with redundant synthetic combinations, and 3. it may explain the unusually high performance metrics that seem inconsistent with the complexity of AD diagnosis.

- Different brain regions have distinct molecular profiles and assuming AD signatures are identical across regions lacks support. This may explain the substantial performance drop in external validation.

- The results require validation in larger, independent studies before drawing clinical conclusions. The feature selection validation using the same data for both identification and testing risks overfitting.

- Several technical aspects need clarification: the "masking mechanism" mentioned in ablation studies is never properly explained, the exact implementation of your synthetic pairing during cross-validation is unclear, and batch harmonization details are insufficient for reproducibility. The sample size discrepancies between your described 80/20 split and the reported table values need resolution.

Response:
We sincerely thank the reviewer for the precise and critical evaluation. These comments prompted us to substantially expand our experimental validation and methodological justification. In response, we address the following key points:
(1) regarding the concern of "artificial relationships," we clarify that the Cartesian pairing strategy is designed to learn population-level features rather than individual-level molecular correspondences, and we further validate its discriminative power using real paired data (AUC = 0.93);
(2) regarding "data inflation," we emphasize that the pairing strategy aims to construct cross-modal statistical associations rather than merely increasing sample size, and that the model relies on stable attention weights across multiple combinations to focus on consistent

biological signals while reducing redundancy;
(3) regarding "inflated performance," we explain that the high internal accuracy arises from the strong label consistency of the autopsy-based molecular classification task, while the performance decrease observed in cross-brain-region and cross-batch validations (AUC = 0.84) is expected given the known heterogeneity of Alzheimer's disease, and that the interpretable features remain consistent with established pathological pathways;
(4) regarding the "cross-brain-region assumption," we provide a newly added comparison of top-ranked features between frontal and temporal cortex datasets, demonstrating that the model automatically captures region-specific markers without enforcing feature consistency, and that the observed performance drop in external validation reflects genuine regional molecular differences;
(5) regarding "independent validation and overfitting," we introduce an additional independent cohort comprising 181 transcriptomic samples and 147 methylation samples from the same brain region (AUC = 0.94), as well as a real paired cohort (AUC = 0.93), providing further evidence against overfitting. Detailed point-by-point responses and newly added results are provided below.

Reviewer #2, Comment 1

Creating all possible combinations between RNA-AD samples and Methylation-AD samples fundamentally violates the biological reality that each patient has unique cross-modal molecular relationships. This synthetic pairing strategy assumes all AD patients share identical RNA-methylation associations, which is biologically implausible given the known heterogeneity of Alzheimer's disease.
This approach creates several problems:

1. it generates artificial relationships that don't exist in real patients.

Response:

We sincerely thank the reviewer for this fundamental and highly insightful comment. We fully agree that, from the perspective of precise molecular regulation, cross-omics relationships within an individual are highly specific, and that our pairing strategy cannot reconstruct such individual-level biological relationships. We therefore clarify that this approach represents a pragmatic compromise under the constraint of unpaired data, rather than an attempt to model individual-specific regulation. Similar strategies have been adopted in prior studies integrating unpaired multi-omics data [2], with their effectiveness validated across multiple applications [3–5].

Importantly, the design goal of our method is not to capture individual-specific regulatory mechanisms, but rather to learn population-level cross-omics functional modules that are statistically associated with disease status. By pairing all samples sharing the same label, the model is exposed to diverse molecular subtypes of Alzheimer's disease, thereby implicitly

modeling disease heterogeneity and learning a more robust and representative cross-omics association landscape.

Moreover, multiple components of our framework act as safeguards against learning spurious associations. The multi-head attention mechanism of the Transformer and the bidirectional reconstruction loss together serve as strong regularizers, enabling the model to down-weight inconsistent co-occurrences and focus on cross-modal relationships that are both predictive and biologically coherent.

Most importantly, the strongest evidence for the validity of this strategy comes from empirical evaluation on real paired data. When the trained model was applied to a genuinely paired frontal cortex dataset (GSE110732; 25 paired samples), it achieved an accuracy of 0.88 (Table 2; Figure 4) and an AUC of 0.93 (Table 2; Figures 4 and 5), which is comparable to its performance on the internal test set.

Taken together, these results indicate that, despite its theoretical limitations, the proposed pairing strategy can effectively extract biologically meaningful and generalizable cross-omics features from unpaired data. This provides a practical and necessary solution for multi-omics integration under realistic data constraints, particularly when the primary objective is disease classification rather than individual-level mechanistic inference.

Table 2: Performance evaluation of the models on internal and external test sets

DataSet	Accuracy	Precision	Recall	F1	AUC
Test	0.9736	0.9788	0.9803	0.9796	0.9910
External test1	0.7389	0.6901	0.8884	0.7768	0.8432
External test2	0.8732	0.7925	0.9962	0.8827	0.9396
Paired data test	0.8800	0.7692	1.0	0.8696	0.9267

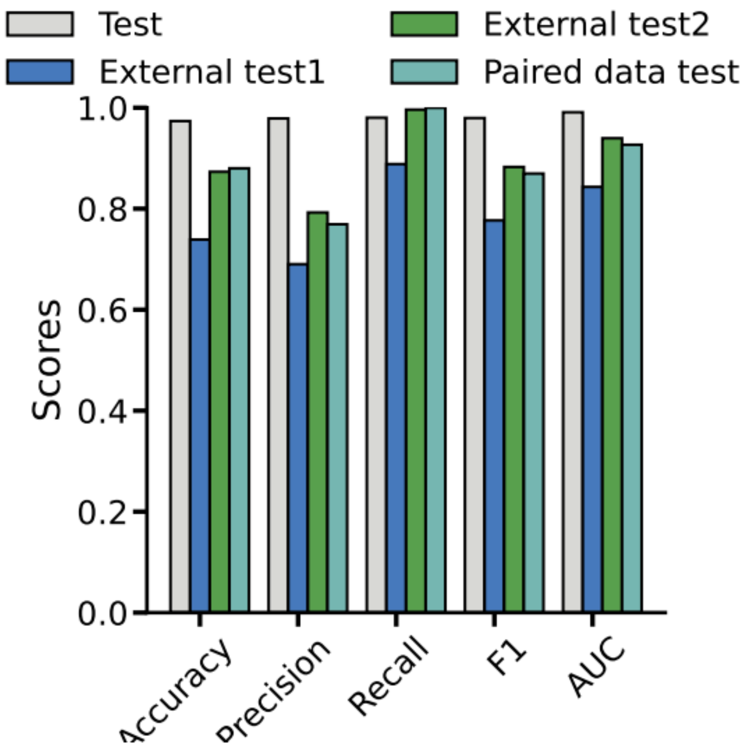

Figure 4. Bar-plot performance comparison of the model on the internal test set and external test sets (cross-region, same-region, and paired datasets)

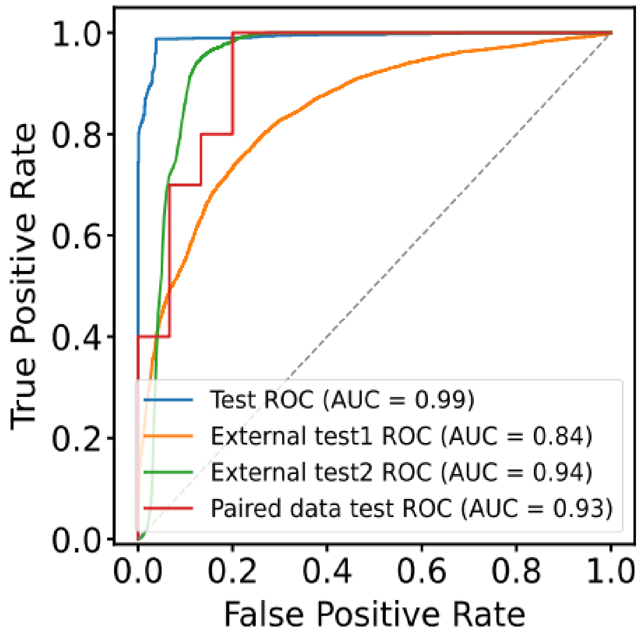

Figure 5. AUC curves of the model on the internal test set and external test sets (cross-region, same-region, and paired datasets)

2. it dramatically inflates your dataset size with redundant synthetic combinations, and
We fully understand the reviewer's concern that synthetic pairing strategies may introduce data redundancy, and we sincerely appreciate this valuable comment. Here, we would like to clarify the original motivation and the actual effect of this design choice.

First, the combinatorial pairing strategy adopted in our study is essentially a biologically informed cross-modal association enhancement approach tailored for unpaired multi-omics data. The core objective of this strategy is not to artificially increase the effective sample size, but rather to systematically construct RNA–methylation sample pairs under the same disease label, thereby enabling the model to learn population-level cross-modal association patterns that transcend individual-level heterogeneity. This enhancement strategy directly addresses a fundamental challenge in zero-pairing scenarios, where models otherwise lack any supervision to learn cross-modal relationships.

Second, the model architecture itself provides intrinsic mechanisms to mitigate redundancy. Specifically, the multi-head attention mechanism and the bidirectional reconstruction loss act jointly as strong regularizers, allowing the model to automatically suppress individual-specific noise and focus on cross-modal associations that consistently recur across multiple combinations. As a result, the model preferentially learns stable and biologically meaningful relationships rather than spurious correlations induced by artificial pairing.

We fully agree that fully paired datasets are ideal. However, under the realistic constraint of limited paired multi-omics data, this disease-informed association enhancement strategy offers a practical and effective alternative for cross-modal learning. We sincerely thank the reviewer for this insightful comment and welcome further guidance.

3. it may explain the unusually high performance metrics that seem inconsistent with the complexity of AD diagnosis.

We thank the reviewer for this thoughtful observation regarding model performance in the context of the inherent complexity of Alzheimer's disease (AD). We fully agree that AD diagnosis is highly complex, which is precisely why early diagnosis remains challenging.

First, the relatively high performance achieved by our model primarily reflects the nature of the task it addresses: a molecular classification problem based on postmortem brain tissue, distinguishing confirmed AD cases from controls. Compared with in vivo clinical diagnosis at preclinical stages, this task is intrinsically less ambiguous. These rigorously quality-controlled cohorts provide highly consistent molecular labels, allowing the model to learn clearer discriminative patterns.

Second, within homogeneous internal cohorts, the model demonstrates its theoretical potential by learning consistent disease-associated molecular features. In contrast, during external validation across different brain regions, technical batches, and patient subtypes, performance shows a reasonable and expected decline, which aligns well with the known biological

heterogeneity of AD. Importantly, this decline should not be interpreted as a model deficiency, but rather as a realistic reflection of underlying biological variability.

Furthermore, interpretability analyses reveal that the features driving model predictions are enriched in pathways known to be involved in AD pathology, providing additional biological support for the validity of the learned representations.

We sincerely appreciate the reviewer's cautious perspective, which has motivated us to more rigorously contextualize our results. We believe that the strong internal performance reflects effective cross-modal integration, while the behavior observed in external validation faithfully mirrors the intrinsic complexity of AD biology.

Reviewer #2, Comment 2

Different brain regions have distinct molecular profiles and assuming AD signatures are identical across regions lacks support. This may explain the substantial performance drop in external validation.

Response:

We are very grateful to the reviewer for this highly insightful explanation, and we fully agree with this assessment. The observation that different brain regions possess distinct molecular characteristics provides a compelling biological explanation for the reasonable performance decline observed in cross-region external validation. Notably, the recovery of performance on newly added external datasets from the same brain region strongly supports this interpretation and reflects the biological reality of Alzheimer's disease (AD).

Our motivation for performing cross-region validation was precisely to conduct such a stringent stress test. Encouragingly, despite substantial regional heterogeneity, the model still achieved a robust performance (AUC = 0.8432). Thus, the transition from very high internal performance to stable but lower external performance does not indicate overfitting; rather, it highlights the impact of true biological variability.

To further substantiate this point, we additionally evaluated the model on frontal cortex datasets. The unpaired external dataset includes 181 transcriptomic samples (GSE5281 [3], GSE15222 [4], GSE12685 [5], GSE36980 [6], GSE48350 [7]) and 147 methylation samples (GSE59685 [8], GSE66351 [9]). The paired dataset (GSE110732 [10]) contains 25 transcriptomic and 25 methylation samples. After preprocessing, the unpaired dataset comprised 116 AD cases and 65 controls, while the paired dataset included 10 AD cases and 15 controls.

On the unpaired frontal cortex dataset, the model achieved an Accuracy of 0.88 (Table 2, Fig. 4) and an AUC of 0.94 (Table 2, Fig. 4). On the paired dataset, the Accuracy and AUC were

0.88 and 0.93, respectively. These results represent a clear improvement compared with those obtained on the superior temporal gyrus dataset, thereby directly validating the reviewer's hypothesis and demonstrating the model's robustness under biologically favorable conditions.

To further investigate this phenomenon, we compared important features identified by Integrated Gradients between the internal training set (frontal cortex) and the external test set (superior temporal gyrus). The top 20 features differed substantially between the two brain regions. For the frontal cortex dataset, the top features included MS4A7, ATXN2, XPO6, Methy_PCDHA13, GART, WDR48, MAB21L2, SNORD8, TPK1, ADD1, RPS10, Methy_MEF2C, GNPDA1, Methy_PRKD3, MRPL39, MLKL, MUPCDH, PLEKHA9, and C11orf60. In contrast, the superior temporal gyrus dataset highlighted MS4A7, Methy_MS4A2, Methy_UQCC, TBC1D1, RPL35, Methy_SMPD3, CKB, RPL27, Methy_NCK2, WDR48, MEF2C, RPL41, PRKD3, BLZF1, RPS10, TPK1, CLU, ATP5O, and TPD52L1.

As illustrated in Figure 6, the substantial divergence between the top features across brain regions further corroborates the reviewer's insight and confirms that the model does not impose a false assumption of cross-region molecular consistency.

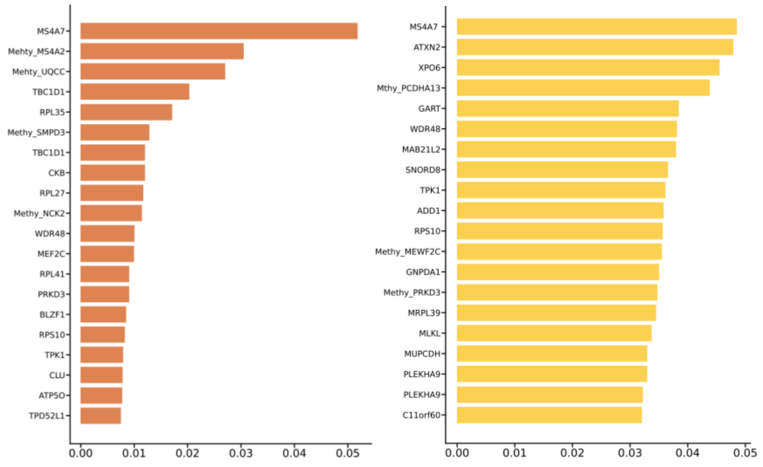

Figure 6. Important features extracted by the explainability module on the external cross-region dataset from the temporal cortex (left) and the same-region dataset from the frontal cortex (right)

In summary, by systematically comparing model performance across different brain regions and examining the top 20 important features identified in each region, we demonstrate that both predictive performance and feature attribution indeed vary across brain regions. The model attends to distinct sets of molecular features depending on the brain region analyzed, which is most likely attributable to region-specific molecular characteristics. We sincerely thank the reviewer for helping us articulate this point more clearly, which has significantly strengthened the rigor and biological plausibility of our argument.

Reviewer #2, Comment 3

The results require validation in larger, independent studies before drawing clinical conclusions. The feature selection validation using the same data for both identification and testing risks overfitting.

Response:

We sincerely appreciate this crucial comment. Model robustness and generalizability are fundamental prerequisites for any potential clinical application, and avoiding overfitting during feature selection is a key component of this process.

(1) Validation on larger and independent datasets

In response to the reviewer's suggestion, we have incorporated two additional external datasets. The model achieved an AUC of 0.94 on the unpaired external dataset and an AUC of 0.93 on the paired external dataset, as detailed below.

We acknowledge that acquiring large-scale, fully independent Alzheimer's disease cohorts with paired multi-omics measurements remains a major challenge in the field and represents a common bottleneck for many studies. Nevertheless, within the limits of currently available resources, we have made substantial efforts to rigorously assess the generalizability of our model.

Specifically, we directly applied the trained AE-Trans model to a newly collected external dataset comprising 181 transcriptomic samples and 147 DNA methylation samples from the same brain region as the training data. On this cohort, the model achieved robust performance (AUC = 0.94; Table 2, Fig. 4), providing evidence that its predictive capability is not confined to the training data distribution. In addition, we evaluated the model on a real-world paired dataset and obtained similarly strong performance (AUC = 0.93; Table 2, Fig. 4). Together, these results strongly suggest that AE-Trans learns generalizable latent representations rather than overfitting to dataset-specific noise or feature combinations.

(2) Addressing the risk of overfitting in feature selection

We fully agree that the risk of overfitting in feature selection is an important concern, and we appreciate the reviewer's precise observation. Here, we clarify our workflow to demonstrate how we have mitigated this risk.

First, the counterfactual integrated gradients method used to identify important features was applied after model training, with all model parameters fixed. This procedure constitutes a post hoc interpretability analysis rather than an iterative feature selection process during model optimization. Model weights were learned solely on the training data, while feature importance scores were computed across all samples and subsequently averaged to obtain a stable ranking.

Second, to directly validate the predictive value of the identified features, we conducted a critical downstream experiment: we retrained a logistic regression classifier using only the top 200 features identified by AE-Trans and evaluated its performance on an independent test set. This classifier achieved a high AUC of 0.9749 (Fig. 4 in the original manuscript). This experiment is particularly important because it demonstrates that the identified features themselves possess strong predictive power that transfers to a simpler and entirely different model, and remains robust on unseen data. This substantially reduces the likelihood that the feature importance scores merely reflect overfitting arising from complex model–feature interactions.

We fully concur with the reviewer that definitive clinical inference ultimately requires validation in large-scale, prospective, real-world cohorts. Accordingly, we explicitly acknowledge this as a key direction for future work. While the present study provides initial evidence of generalizability through multiple external datasets and downstream validation tasks, further confirmation in larger, multi-center, prospectively collected Alzheimer's disease cohorts will be essential to establish clinical utility.

Once again, we sincerely thank the reviewer for this valuable feedback, which has prompted us to more clearly articulate both the limitations of the current study and its future directions. We hope that the above explanations and supporting evidence demonstrate that we are fully aware of these limitations and have adopted multiple feasible strategies under current data constraints to evaluate and enhance model robustness, thereby laying a solid foundation for future translational research.

Reviewer #2, Comment 4

- Several technical aspects need clarification: the "masking mechanism" mentioned in ablation studies is never properly explained, the exact implementation of your synthetic pairing during cross-validation is unclear, and batch harmonization details are insufficient for reproducibility. The sample size discrepancies between your described 80/20 split and the reported table values need resolution.

（1）the "masking mechanism" mentioned in ablation studies is never properly explained

Response:

We thank the reviewer for pointing out the lack of a formal description of the masking mechanism and provide a detailed clarification here. In our model, we introduce a feature-level random masking mechanism as a key regularization and data augmentation strategy. This mechanism is directly integrated at the input layer of the model. For each mini-batch during training, we randomly select positions from the 10,000-dimensional input feature vector and forcibly set the corresponding feature values to zero.

This operation deliberately simulates scenarios of partial feature absence in the original data. The primary motivation is to encourage the model to learn effective representations and patterns from incomplete and partially occluded inputs, rather than relying on a small subset of dominant features. Such a design reflects realistic data acquisition conditions, where technical noise or partial measurement failure frequently occurs. By continuously training on these artificially "corrupted" inputs, the model is guided to capture deeper and more robust inter-feature dependencies. As a result, this mechanism effectively mitigates overfitting on the training data and improves the generalization ability and robustness of the model when applied to external datasets that may contain missing or noisy features.

（2）the exact implementation of your synthetic pairing during cross-validation is unclear

Response:

We appreciate the reviewer's comment and provide a detailed description of how the synthetic pairing strategy is implemented within the cross-validation framework. The core principle of this strategy is that synthetic multimodal instances are constructed strictly within each data split, and only among samples belonging to the same class, thereby ensuring strict data isolation and preventing any information leakage. Specifically, in each fold of cross-validation, RNA and methylation datasets are first independently divided into positive (AD) and negative (Control) samples. Each class is then further split into training and validation subsets. Within the training set, RNA samples from the positive class are paired with methylation samples from the positive class in a pairwise (Cartesian product) manner to generate all possible positive multimodal feature pairs. The same procedure is applied to the negative class to generate negative pairs. The validation set follows an identical pairing strategy, using only the independently held-out validation samples. Through this within-class Cartesian pairing across modalities, we construct an expanded multi-modal dataset without introducing additional samples or cross-set contamination. This strategy enables effective multi-modal learning while strictly preserving the integrity of the evaluation protocol.

（3）and batch harmonization details are insufficient for reproducibility

Response:

We thank the reviewer for this valuable suggestion and provide additional details regarding batch effect correction. During the data integration stage, raw data originating from different platforms or experimental batches were corrected using the ComBat algorithm, which is based on an empirical Bayes framework. This approach effectively removes technical variations while preserving disease-related biological signals. The batch-corrected data were saved and used as the direct input for model training.

Subsequently, during the model training stage, feature-level standardization was applied to ensure consistent feature scales and to improve the stability of neural network optimization.

Importantly, all transformation parameters were fitted exclusively on the training data and then applied to the validation and test sets, strictly preventing any form of data leakage. These procedures together ensure that the results are fully reproducible.

（4）The sample size discrepancies between your described 80/20 split and the reported table values need resolution.

Response:

We thank the reviewer for raising this concern and acknowledge that the original description may have caused confusion. We clarify here that the reported 80/20 split refers to the initial partitioning of the original RNA and methylation datasets prior to applying the synthetic pairing strategy, rather than to the composition of the final paired dataset. The sample numbers reported in the tables correspond to the dataset size after synthetic pairing.

Specifically, in the train–test experiments, the RNA dataset contains 697 samples (439 positive and 258 negative). These were split into 351 positive and 206 negative samples for training, and 88 positive and 52 negative samples for testing. The methylation dataset contains 142 samples (74 positive and 68 negative), which were split into 59 positive and 54 negative samples for training, and 15 positive and 14 negative samples for testing. After synthetic pairing, the final training set contained 31,833 samples (351 × 59 + 206 × 54), and the test set contained 2,048 samples (88 × 15 + 52 × 14).

For cross-validation, the RNA training set was further divided into 281 positive and 164 negative samples for training, and 70 positive and 41 negative samples for validation. The methylation training set was divided into 47 positive and 43 negative samples for training, and 12 positive and 11 negative samples for validation. After pairing, this resulted in 20,259 samples in the training set (281 × 47 + 164 × 43) and 1,302 samples in the validation set (70 × 12 + 42 × 11).

REFERENCES

1. Ji, Z., et al., *Raformer: Redundancy-Aware Transformer for Video Wire Inpainting.* IEEE Trans Image Process, 2025. **34**: p. 1795-1809.
2. Wang, J., et al., *A semi-supervised approach for the integration of multi-omics data based on transformer multi-head self-attention mechanism and graph convolutional networks.* BMC Genomics, 2024. **25**(1): p. 86.
3. Liang, W.S., et al., *Gene expression profiles in anatomically and functionally distinct regions of the normal aged human brain.* Physiol Genomics, 2007. **28**(3): p. 311-22.

4. Webster, J.A., et al., *Genetic control of human brain transcript expression in Alzheimer disease.* Am J Hum Genet, 2009. **84**(4): p. 445-58.
5. Celia Williams., et al., *Transcriptome Analysis of Synaptoneurosomes Identifies Neuroplasticity Genes Overexpressed in Incipient Alzheimer's Disease.* PLoS ONE, 2009. **4**(3): p. 1-13.
6. Hokama, M., et al., *Altered Expression of Diabetes-Related Genes in Alzheimer's Disease Brains: The Hisayama Study.* CEREBRAL CORTEX, 2014. **24**(9): p. 2476-2488.
7. Nicole, C.B., et al., *Gene expression changes in the course of normal brain aging are sexually dimorphic.* Proc Natl Acad Sci U S A, 2008. **105**(40): p. 15605-10.
8. Katie Lunnon., et al., *Methylomic profiling implicates cortical deregulation of ANK1 in Alzheimer's disease.* Nat Neurosci, 2014. **17**(9): p. 1164-70.
9. Gilles Gasparoni., et al., *DNA methylation analysis on purified neurons and glia dissects age and Alzheimer's disease-specific changes in the human cortex.* Epigenetics Chromatin, 2018. **11**(1): p. 41.
10. Peipei, L., et al., *Epigenetic dysregulation of enhancers in neurons is associated with Alzheimer's disease pathology and cognitive symptoms.* Nature Communications, 2019. **1**(1): p. 2246.
11. Ignazio S Piras., et al., *Transcriptome Changes in the Alzheimer's Disease Middle Temporal Gyrus: Importance of RNA Metabolism and Mitochondria-Associated Membrane Genes.* J Alzheimers Dis, 2019. **70**(3): p. 691-713.
12. Patel, H., et al., *Transcriptomic analysis of probable asymptomatic and symptomatic alzheimer brains.* Brain, Behavior, and Immunity, 2019. **80**(0): p. 644-656.
13. Jeremy A Miller., et al., *Genes and pathways underlying regional and cell type changes in Alzheimer's disease.* Genome Med, 2013. **5**(5): p.48.
14. Zhimeng, Y., et al., *Age-related decline in hippocampal tyrosine phosphatase PTPRO is a mechanistic factor in chemotherapy-related cognitive impairment.* JCI insight, 2023. **8**(14): p. e166306.
