# Supplementary material for: Interpretable integration of unpaired multi-omics for Alzheimer’s diagnosis via cross-modal transformer reconstruction
Source: PLoS Comput Biol. 2026 Mar 12;22(3):e1014074. doi: 10.1371/journal.pcbi.1014074 (PMC12994821; doi:10.1371/journal.pcbi.1014074)
Supplement: Attachment — Submitted filename: Response to Reviewers.docx [file pcbi.1014074.s005.docx]

**Additional Editor Comment:**

I agree with R2 that the authors should add some additional caveats regarding the risk of overfitting.

**Response to the Editor**

We sincerely thank the editors for their valuable and constructive suggestions, which helped us clearly identify the key issues of the manuscript and guided the direction of our revisions. We have added a description of the model's risk of overfitting in the Results 1 and Discussion sections(Highlighted yellow). We hope that you will be satisfied with the revised content of the manuscript.

Result 1

**Performance Comparison of AE-Trans with Baseline Methods**

To assess generalizability, we applied the pretrained AE-Trans model to an external test set comprising middle temporal gyrus RNA-seq data from GSE132903 and methylation data from a distinct cortical region (GSE80970 subset). No further training was performed. First, we tested AE-Trans on a cross-region unpaired dataset, which included RNA-seq data from GSE132903 and methylation data from the superior temporal gyrus in GSE80970, representing data from different brain regions. The model maintained strong performance (External test1; Accuracy = 0.7389, AUC = 0.8432), outperforming DBN by 2.2% in Accuracy and LR by 5.5% in AUC (Table 1; Fig. 2A–B). Notably, all features and thresholds used for internal evaluation were retained during external testing, ensuring comparability. It is worth noting that external dataset is derived from different brain regions, which may increase the risk of model overfitting. Therefore, when making predictions across brain regions, we need to pay special attention to adjusting the model parameters. Next, we validated the model on an unpaired same-region dataset from the frontal cortex, comprising 181 transcriptomes (GSE5281, GSE15222, GSE12685, GSE36980, GSE48350) and 147 methylomes (GSE59685, GSE66351). AE-Trans demonstrated strong generalization with an accuracy of 0.8732 and an AUC of 0.9396, confirming its ability to generalize within the same brain region (External test2; Figure 2E; Table 2).Finally, to assess the model's ability to handle paired data, we tested it on a real-world paired dataset from the frontal cortex (GSE110732), which contained 25 matched transcriptome and methylation samples. AE-Trans achieved excellent results (Accuracy = 0.8800, AUC = 0.9267), showcasing its effectiveness in transferring knowledge from unpaired datasets to paired data (Paried dataset; Figure 2E; Table 2). In contrast to the cross-brain region dataset External test1, the within-brain region external validation set and the genuine paired data demonstrate a relatively lower risk of overfitting.

Together, these results demonstrate that AE-Trans not only excels in classification tasks under matched conditions but also generalizes reliably across anatomical regions and datasets—validating its utility in real-world multi-omics applications for Alzheimer’s disease. When dealing with such studies, we should pay attention to the differences in data sets and adjust parameters to reduce the risk of overfitting.

**Discussion and conclusion**

Alzheimer’s disease (AD) is a complex neurodegenerative disorder that remains difficult to diagnose, particularly at early stages. In this study, we developed AE-Trans, a dual-channel Transformer-based framework capable of integrating unpaired RNA and DNA methylation data. Through multi-stage representation learning and bidirectional reconstruction, AE-Trans effectively captures complementary modality-specific signals and aligns latent features across data types. Our model consistently outperformed comparative methods across a range of classification tasks, including same-brain-region, cross-brain-region, and real paired datasets. Specifically, AE-Trans achieved an AUC of 0.9910 on the prefrontal cortex data, and reached an AUC of 0.8432 on cross-region temporal cortex data. Moreover, it attained an AUC of 0.94 on an external unpaired dataset from the same brain region, and achieved an AUC of 0.93 on a real-world paired multi-omics dataset.. These results demonstrate the robustness and generalizability of our approach for AD classification. However, when conducting such studies, we must pay attention to the variations in datasets across different brain regions to minimize the risk of overfitting that might be introduced.

**Reviewer #2**

 Thank you for addressing the reviewers' comments. The risk of overfitting remains a concern given the very large model size relative to the available data. Although the added external datasets and experiments are helpful, a more cautious interpretation of the results and claims would be advised.

**Response:**
We sincerely thank the reviewer for the precise and critical evaluation. These comments prompted us to substantially expand our experimental validation and methodological justification. In response, We have added a description of the model's risk of overfitting in the Results 1 and Discussion sections(Highlighted yellow). We hope that you will be satisfied with the revised content of the manuscript.

Result 1

**Performance Comparison of AE-Trans with Baseline Methods**

To assess generalizability, we applied the pretrained AE-Trans model to an external test set comprising middle temporal gyrus RNA-seq data from GSE132903 and methylation data from a distinct cortical region (GSE80970 subset). No further training was performed. First, we tested AE-Trans on a cross-region unpaired dataset, which included RNA-seq data from GSE132903 and methylation data from the superior temporal gyrus in GSE80970, representing data from different brain regions. The model maintained strong performance (External test1; Accuracy = 0.7389, AUC = 0.8432), outperforming DBN by 2.2% in Accuracy and LR by 5.5% in AUC (Table 1; Fig. 2A–B). Notably, all features and thresholds used for internal evaluation were retained during external testing, ensuring comparability. It is worth noting that external dataset is derived from different brain regions, which may increase the risk of model overfitting. Therefore, when making predictions across brain regions, we need to pay special attention to adjusting the model parameters. Next, we validated the model on an unpaired same-region dataset from the frontal cortex, comprising 181 transcriptomes (GSE5281, GSE15222, GSE12685, GSE36980, GSE48350) and 147 methylomes (GSE59685, GSE66351). AE-Trans demonstrated strong generalization with an accuracy of 0.8732 and an AUC of 0.9396, confirming its ability to generalize within the same brain region (External test2; Figure 2E; Table 2).Finally, to assess the model's ability to handle paired data, we tested it on a real-world paired dataset from the frontal cortex (GSE110732), which contained 25 matched transcriptome and methylation samples. AE-Trans achieved excellent results (Accuracy = 0.8800, AUC = 0.9267), showcasing its effectiveness in transferring knowledge from unpaired datasets to paired data (Paried dataset; Figure 2E; Table 2). In contrast to the cross-brain region dataset External test1, the within-brain region external validation set and the genuine paired data demonstrate a relatively lower risk of overfitting.

Together, these results demonstrate that AE-Trans not only excels in classification tasks under matched conditions but also generalizes reliably across anatomical regions and datasets—validating its utility in real-world multi-omics applications for Alzheimer’s disease. When dealing with such studies, we should pay attention to the differences in data sets and adjust parameters to reduce the risk of overfitting.

**Discussion and conclusion**

Alzheimer’s disease (AD) is a complex neurodegenerative disorder that remains difficult to diagnose, particularly at early stages. In this study, we developed AE-Trans, a dual-channel Transformer-based framework capable of integrating unpaired RNA and DNA methylation data. Through multi-stage representation learning and bidirectional reconstruction, AE-Trans effectively captures complementary modality-specific signals and aligns latent features across data types. Our model consistently outperformed comparative methods across a range of classification tasks, including same-brain-region, cross-brain-region, and real paired datasets. Specifically, AE-Trans achieved an AUC of 0.9910 on the prefrontal cortex data, and reached an AUC of 0.8432 on cross-region temporal cortex data. Moreover, it attained an AUC of 0.94 on an external unpaired dataset from the same brain region, and achieved an AUC of 0.93 on a real-world paired multi-omics dataset.. These results demonstrate the robustness and generalizability of our approach for AD classification. However, when conducting such studies, we must pay attention to the variations in datasets across different brain regions to minimize the risk of overfitting that might be introduced.
